# Oral and anal high-risk human papilloma virus infection in HIV-positive men who have sex with men over a 24-month longitudinal study: complexity and vaccine implications

**DOI:** 10.1186/s12889-019-7004-x

**Published:** 2019-05-28

**Authors:** Saverio Giuseppe Parisi, Monica Basso, Renzo Scaggiante, Samantha Andreis, Carlo Mengoli, Mario Cruciani, Claudia Del Vecchio, Nicola Menegotto, Daniela Zago, Loredana Sarmati, Massimo Andreoni, Giorgio Palù

**Affiliations:** 1 Department of Molecular Medicine, University of Padova, Via Gabelli 63, 35100 Padova, Italy; 2 Infectious Diseases Unit, Padova Hospital, Via Giustiniani, 2 -, 35128 Padova, Italy; 3 Center of Diffusive Diseases, ULSS 9, Via Campania 1, 37136 Verona, Italy; 4 Clinical Infectious Diseases, Tor Vergata University, Viale Oxford, 81, 00133 Rome, Italy

**Keywords:** High risk HPV, Men who have sex with men, Anal, Oral, HIV infection

## Abstract

**Background:**

Few studies focused on longitudinal modifications over time of high-risk HPV (HR-HPV) at anal and oral sites in HIV+ men who have sex with men (MSM).

**Methods:**

We described patterns and longitudinal changes of HR-HPV detection and the prevalence of HR-HPV covered by the nonavalent HPV vaccine (vax-HPV) at oral and anal sites in 165 HIV+ MSM followed in an Italian hospital. The samples were collected at baseline and after 24 months (follow-up). The presence of HPV was investigated with Inno-LiPA HPV Genotyping Extra II.

**Results:**

Median age was 44 years (IQR 36–53), median CD4+ cell count at nadir was 312 cells/mm^3^ (IQR 187–450). A total of 120 subjects (72.7%) were receiving successful antiretroviral therapy (ART). At baseline and follow-up, the frequency of HR-HPV was significantly higher in the anal site (65.4% vs 9.4 and 62.4% vs 6.8%, respectively). Only 2.9% of subjects were persistently HR-HPV negative at both sites. All oral HR-HPV were single at baseline vs 54.6% at baseline at the anal site (*p* = 0.005), and all oral HR-HPV were single at follow-up vs 54.4% at anal site at follow-up (*p* = 0.002). The lowest rate of concordance between the oral and anal results was found for HR-HPV detection; almost all HR-HPV positive results at both anal and oral sites had different HR-HPV.The most frequent HR-HPV in anal swabs at baseline and follow-up were HPV-16 and HPV-52.At follow-up at anal site, 37.5% of patients had different HR-HPV genotypes respect to baseline, 28.8% of subjects with 1 HR-HPV at baseline had an increased number of HR-HPV, and patients on ART showed a lower frequency of confirmed anal HR-HPV detection than untreated patients (*p* = 0.03) over time. Additionally,54.6 and 50.5% of patients had only HR-vax-HPV at anal site at baseline and follow-up, respectively; 15.2% had only HR-vax-HPV at baseline and follow-up.

**Conclusions:**

We believe that it is important testing multiple sites over time in HIV-positive MSM. ART seems to protect men from anal HR-HPV confirmed detection. Vaccination programmes could reduce the number of HR-HPV genotypes at anal site and the risk of the first HR-HPV acquisition at the oral site.

**Electronic supplementary material:**

The online version of this article (10.1186/s12889-019-7004-x) contains supplementary material, which is available to authorized users.

## Background

Patients with HIV infection are more likely to experience coinfection with human papillomavirus (HPV) compared to HIV negative subjects. Within the HIV positive population, over 80% of men who have sex with men (MSM) were HPV-positive in the anal canal, being higher than 80% and with a high frequency (ranging from 70 to 90%) of high risk HPV (HR-HPV) in most studies [1, 2]. The rates of oral mucosa infection are lower: a value of 28.9% for the pooled prevalence of any type of HPV and of 16.5% for the pooled prevalence of HR-HPV was reported in a random effects meta-analysis and meta-regression on prevalence estimated on 11 studies including 1886 HIV positive MSM [3]. All studies but one [4] explored factors associated with any type of HPV infection; in the multivariate analyses, median age remained a significant contributor to the heterogeneity of any HPV prevalence, after adjusting for HIV status.

The persistence of anal HPV DNA infection plays a central role in carcinogenesis. Marra et al. [5] reported that only anal HPV DNA persistence was independently associated with anal high-grade squamous intraepithelial lesions both in general and by concordant causative HPV-type in a cohort of HIV-positive MSM with a mean nadir CD4 T-cell count of 245 ± 134 cells/mm^3^. Limited evidence-based data on the independent role of long-term HPV DNA positivity on the incidence of head and neck squamous cell carcinoma in HIV+ MSM are available [6].

However, negative HPV detection does not imply definite clearance of the infection because the patient’s immune system may be able to control the infection below the detection limit of available HPV assays [7]. Furthermore, testing different sites of the same subject can show different results that are not associated with one another [8], and HPV-DNA positivity rates are higher in HIV positive MSM with respect to HIV negative men at external genital sites, the anal canal and the oral cavity [9]. Therefore, longitudinal studies including the testing of at least 2 anatomic sites is useful to properly identify the HPV status of patients.

The aim of this work was to analyze the numerosity and the confirmed detection of oral and anal HPV infection over a 24-month period in a cohort of HIV-positive MSM; we focused on pattern and longitudinal changes in HR-HPV detection and the prevalence of HPV genotypes covered by the nonavalent HPV vaccine.

## Methods

### Study design

HIV-positive MSM treated as outpatients at the Infectious Disease Unit of Padova University Hospital (Italy) from January 2013 to December 2013 were consecutively invited to take part to this study.

The following inclusion criteria were applied: 1) aged > 18 years; 2) no cutaneous and/or mucosal lesion due to HPV infection at study entry and in the past; 3) no previous HPV vaccination; and 4) able to make the decision to sign the informed consent. Anal HPV testing was included in their clinical workflow: they were asked to give the consent for testing also oral mucosa and for the use of their blinded data. No payment was provided to the subjects.

HPV was tested in oral and anal swabs. All enrolled patients were evaluated by a physician and the samples were collected on two different visits separated by a 24-month interval (the maximum spread was 2 weeks before or after the deadline). The study time corresponding to the first sampling was defined as baseline and the time corresponding to the second sampling was defined as follow-up.

The treating physician decided to start antiviral therapy or to left the patients untreated as prescribed by international guidelines that were current when the study started [10]. The subjects were defined with undetectable plasma HIV viremia when no HIV RNA determination higher than 50 copies/ml was obtained during the entire study period; the detection of one viral blip (defined as a single plasma HIV RNA value between 50 and 500 copies/ml followed by a viral load not detectable) a year was acceptable [11]. The definition of detectable HIV viremia included any other result [12].

The variables studied at baseline were age in years at baseline, the achievement of plasma HIV RNA suppression throughout the study duration and CD4 lymphocyte count at nadir (cells/μl).

The Ethics Committee of Padova University Hospital (prot. 2606-12P) approved this study, which was made according to the Declaration of Helsinki and local legislation. The patients included in the study gave written informed consent to all procedures and they agreed with the anonymous use of their data for scientific aims and publication.

### Specimen testing

At baseline and at follow-up, anal Dacron swab PBS (phosphate-buffered saline)-moistened was inserted for 3 cm length into the anal canal, then the scraping of anal walls was made, rotating clockwise and anticlockwise three times each way. Oropharyngeal mucosa was scraped with strength to obtain oral samples by rotating twice clockwise and twice anticlockwise with another swab. After, the samples were agitated in 1.5 mL of 1 × PBS. The samples were stored at 4 °C and analyzed after no more than 4 h. The QIAamp DNA Mini Kit (Qiagen, GmbH, Hilden, Germany) was used for total DNA extraction: after, primers PC04 and GH20 (which target a 268-bp fragment of beta-globin) were first used. The samples were always processed and stored separately.

No further analysis was performed on samples negative for beta-globin. ExoSAP-IT (USB Corporation, Cleveland, OH) was used to purify the DNA and Inno-LiPA HPV Genotyping Extra II (Fujirebio Europe N.V., Gent, Belgium) [13] was the method chosen to investigate the presence of HPV DNA. All HPV types included in INNO-Lipa assay were tested. We did not include the detailed data in the study because we focused on HR-HPV. The HPV genotype risk level was categorized according to the criteria defined by the International Agency for Research on Cancer: HR-HPV types with sufficient evidence for causing cancer include HPV-16, − 18, − 31, − 33, − 35, − 39, − 45, − 51, − 52, − 56, − 58 and − 59 [14]. Overall, other HPV genotypes and non typeable HPV were considered not high risk (non-HR).

### Statistical analysis

The statistical analysis was performed on all subjects whose valid results were available and on the subgroup of subjects who had HPV infection due to genotypes covered by the nonavalent vaccine (HPV types 16, 18, 31, 33, 45, 52 and 58) [15]. The HPV testing results were defined as HR when one or more HR genotypes were detected, regardless of coinfection with non-HR; the results were defined as non-HR when only non-HR-HPV genotypes were identified in the sample.

Continuous variables were expressed as the median and interquartile range whereas categorical variables were indicated as the absolute number and frequency (%). The Mann-Whitney U test, the chi-squared test and Fisher’s exact test were employed as appropriate.

The significance of the prevalence change from baseline to follow-up was estimated by Pearson’s chi-square test or by Fisher’s exact test, as appropriate.

*P* values < 0.05 were considered to be significant.

## Results

A total of 171 HIV+ MSM responded to the inclusion criteria: 4 subjects had not CD4+ cell count available and 2 subjects refused to provide informed consent, so 165 HIV+ MSM were included in the study. The median age was 44 years (IQR 36–53 years), and the median CD4+ cell count at nadir was 312 cells/mm^3^ (IQR 187–450 cells/mm^3^). Further, 120 subjects (72.7%) were receiving successful ART, and most patients were treated with a protease inhibitor as the third drug (91 of 120, 75.8%).

Valid anal samples were obtained from all patients at baseline and follow-up, whereas valid oral samples were obtained from 106 (64.2%) patients at baseline and from 162 (98.2%) patients at follow-up. Invalid oral samples were not analyzed if they were found negative for beta globin, possibly because of low cellularity. The overall prevalence of HPV (both HR-HPV and non-HR-HPV) in the anal specimens was 89.1% (147/165 patients) at baseline and 89.7% (148/165 patients) at follow-up, and it was significantly lower in the oral specimens (28.3%, 30/106 patients at baseline and 22.8%, 37/162 patients, at follow-up, *p* < 0.0001) HPV detection was confirmed in 12/103 (11.6%) oral samples and in 135/165 anal samples (81.8%).

### Anal and oral HR-HPV detection at baseline and follow-up

**Fig. 1:**
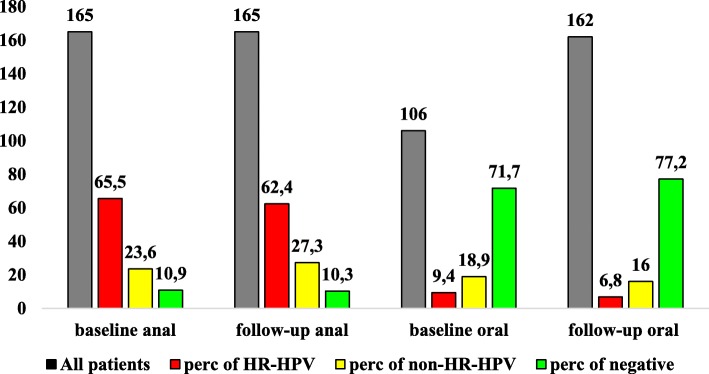
Description of oral and anal swabs results in MSM at baseline and follow-up (24 months after baseline). Data are expressed as absolute numbers (grey column, corresponding to the total number of samples of the specific study time) and as percentage of samples with HR HPV detection, non-HR HPV detection and no HPV detection in the specific study time (red, yellow and green columns). MSM: men who have sex with men. HR-HPV: high risk HPV genotypes. Non-HR-HPV: only non high risk HPV genotypes. HPV: human papillomavirus

At baseline and follow-up, the absolute HR-HPV positivity was significantly higher in anal samples than in oral samples (*p* < 0.0001) whereas, the percentage of HR-HPV detected at anal and oral sites at baseline and at follow-up was comparable (65.4%, 108/165 patients versus 62.4%, 103/165 patients, and 9.4%, 10/106 patients versus 6.8%, 11/162 patients, respectively). The relative frequency of patients with HR-HPV compared to all HPV-positive subjects was significantly higher in anal samples than in oral samples at both baseline (73.5%,108/147 patients versus 33.3% 10/30 patients *p* < 0.0001) and follow-up (69.6%, 103/148 patients versus 29.8%,11/37 patients *p* < 0.0001). A description of the oral and anal swabs results according to HPV detection is reported in Fig. 1.

Overall, 179 anal HR-HPV strains were identified at baseline (strains/person, 1.65) and 194 strains were identified at follow-up (strains/person, 1.88). The most frequent types of HR-HPV detected in the anal swabs at baseline and follow-up were HPV-16 (25.9%, 28/108, and 28.1%, 29/103 of HR-HPV-positive subjects, respectively) and HPV-52 (23.1%, 25/108 and 27.2%, 28/103 of HR-HPV positive subjects, respectively).

All HR positive oral samples at baseline and follow-up had a single HR-HPV detection; the prevalence of this pattern was significantly higher compared to the prevalence in the anal site at baseline (54.6%, 59/108 patients *p* = 0.005) and follow-up (54.4%, 56/103 patients, *p* = 0.002). Among these HR-HPV positive anal samples, multiple HR-HPV patterns were found in 49 of 108 subjects (45.4%) at baseline and in 47 of 103 subjects (45.6%) at follow-up (*p* = 0.0331 with respect to the frequency at baseline) Of note, a different genotype combination was identified in all these patients at baseline and in 90.3% of these patients at follow-up.

**Fig. 2:**
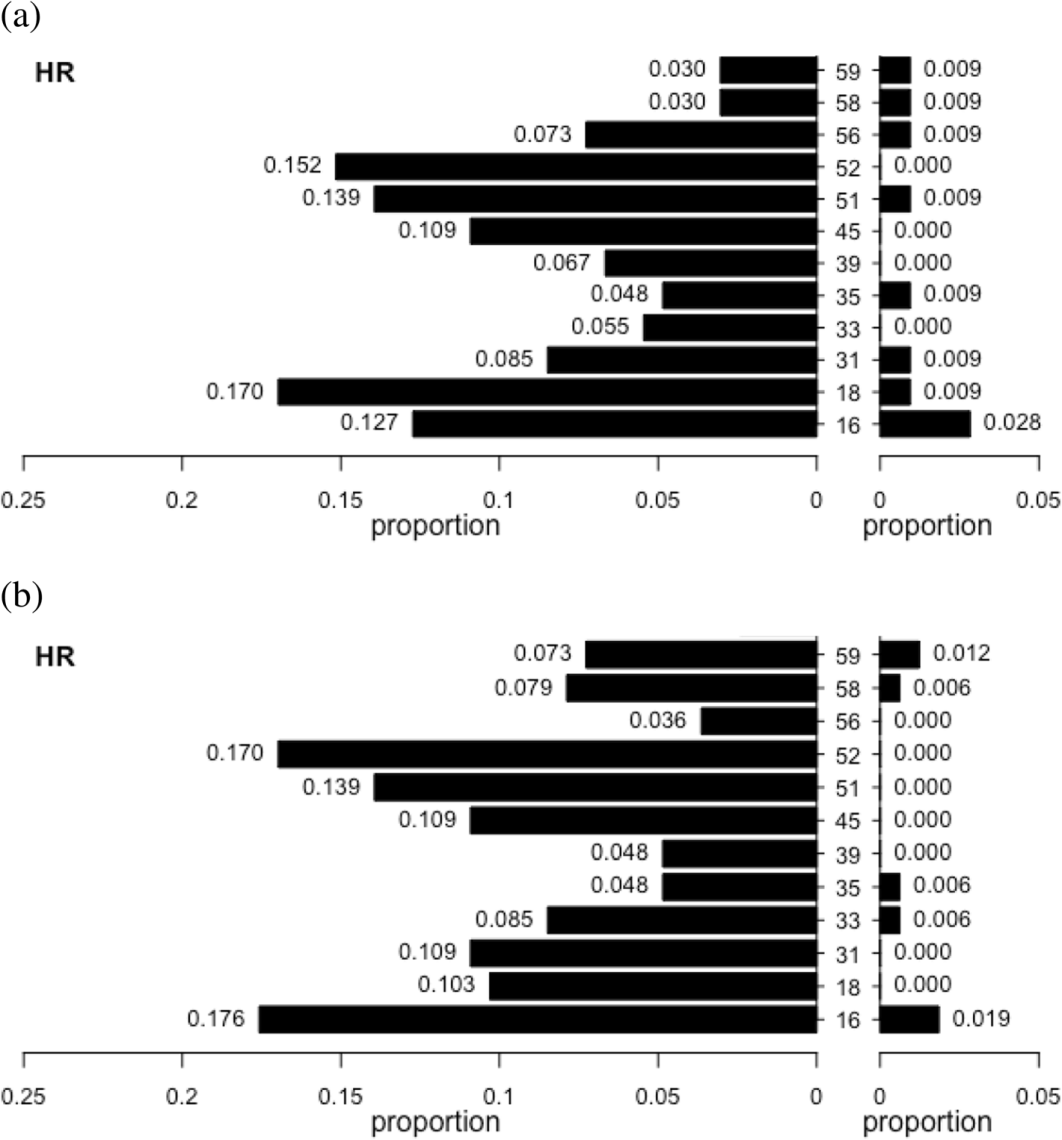
Description of HR-HPV genotype prevalence at baseline (**a**) and at follow-up (24 months after baseline) (**b**). Data corresponding to the anal site are on the left and data corresponding to the oral site are on the right: the prevalence is expressed as proportion respect to the number of subjects with HPV testing available (165 anal samples at baseline and at follow-up, 106 oral samples at baseline and 162 oral samples at follow-up). HR-HPV: high risk HPV genotypes. HPV: human papilloma virus

A complete description of the HR-HPV genotypes and of their single and combined detection in the oral and anal swabs is reported in Fig. 2 and Additional file 1.

### Relationship between oral and anal results at baseline and follow-up

A total of 106 subjects at baseline and 162 patients at follow-up had both oral and anal testing results available. In total, the anal results were concordant with the oral results in 17 patients (16%) at baseline and in 34 patients (21%) at follow-up.

The highest rate of concordance between the oral and anal figure was found for negative results (66.7%, 6/9 patients at baseline and 100%, 17/17 patients at follow-up) and the lowest for HR-HPV detection (9.1%, 7/77 patients at baseline, *p* = 0.0002 and 7.9%, 8/101 patients at follow-up, *p* < 0.0001 with respect to the percentage of concordant negative results at the same study time).

Interestingly, almost all patients with HR positivity at both the anal and oral sites had different HR-HPV genotypes; only a single subject had HPV-16 in both samples at baseline. Table 1 summarizes the combinations between the anal and oral results at baseline and follow-up.

**Table 1:** Description of HPV detection in paired anal and oral swabs baseline and at follow-up (24 months after baseline)

	Baseline oral HR-HPV n (%)	Baseline oral non-HR-HPV n (%)	Baseline oral neg n (%)
Baseline anal HR-HPV (77 pts)	7 (9.1)	15 (19.5)	55 (71.4)
Baseline anal non-HR HPV (20 pts)	1 (5)	4 (20)	15 (75)
Baseline anal neg (9 pts)	2 (22.2)	1 (11.1)	6 (66.7)
	Follow-up oral HR-HPV n (%)	Follow-up oral non-HR-HPV n (%)	Follow-up oral neg n (%)
Follow-up anal HR-HPV (101 pts)	8 (7.9)	17 (16.8)	76 (75.2)
Follow-up anal non-HR-HPV (44 pts)	3 (6.8)	9 (20.5)	32 (72.7)
Follow-up anal neg (17 pts)	0	0	17 (100)

HR: high risk HPV genotypes

non-HR: only not high risk HPV genotypes

HPV: Human papillomavirus

pts: patients

In the subgroup of subjects with all samples tested at both baseline and follow-up, 3 subjects (2.9%) were always positive for HR-HPV at both the anal and oral sites and 3 patients (2.9%) were persistently negative.

### Longitudinal changes in oral and anal testing from baseline to follow-up

A total of 104 patients had HPV tested in oral samples at both baseline and at follow-up. The percentage of patients with the same pattern at both study times was significantly higher in patients with negative results (85.3%, 64/75 patients) with respect to the detection of non-HR-HPV (36.8%, 7/19 patients *p* < 0.0001) and of HR-HPV (40%, 4/10 patients, *p* = 0.003). The HR genotypes of the 4 patients with confirmed HR-HPV at the oral sites were the same at baseline and follow-up (HPV-16, HPV-35, HPV-56, HPV-59).

The number of patients with anal HR-HPV was comparable at baseline and follow-up (108 and 103 respectively), and most (80 out of 108, 74.1%) of the subjects with HR-HPV at baseline were also positive for HR-HPV at follow-up (*p* = 0.032 with respect to the 40% found in oral samples). Patients receiving ART showed a significantly lower frequency of confirmed HR detection compared to untreated patients (43.3%, 52/120 patients versus 62.2%, 28/45 patients, *p* = 0.03). At follow-up, 30 out of 80 patients (37.5%) had HR-HPV at follow-up with a different genotype than that at baseline, 37 patients (46.3%) had one HR-HPV strain with the same genotype at both baseline and follow-up, 11 (13.7%) had 2 HR-HPV and 2 (2.5%) had 3 HR-HPV with the same genotypes at baseline and follow-up.

**Fig. 3:**
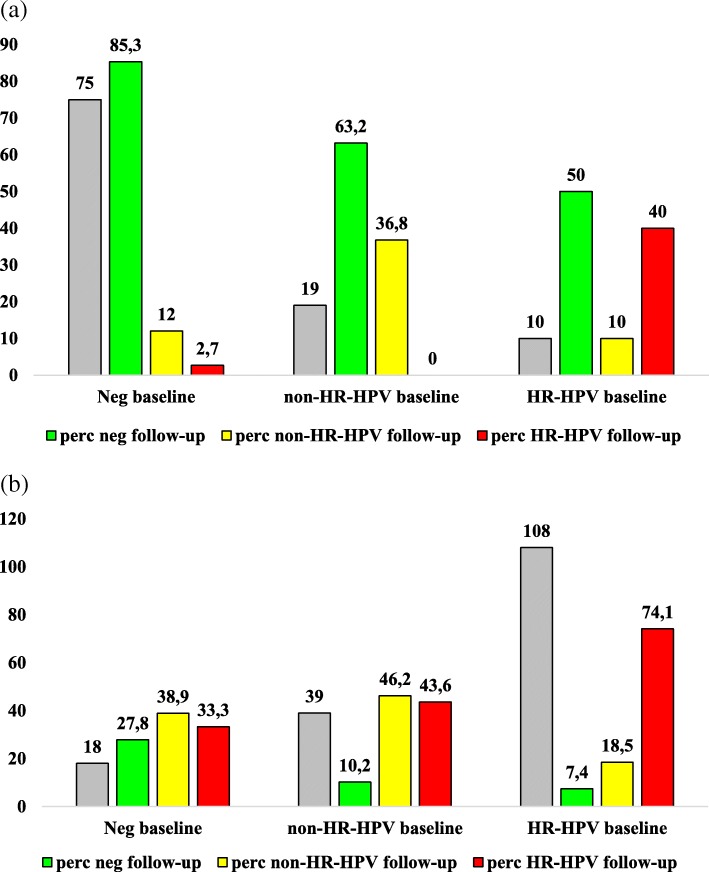
Description of modifications at follow-up (24 months after baseline) of HPV patterns detected at baseline. The first column correspond to baseline data (expressed as absolute number). Green, yellow and red columns correspond to the proportion of negative, non-HR-HPV HR-HPV respectively detected at follow-up as evolution of each HPV pattern at baseline. Panel **a** refers to oral samples and panel **b** refers to anal samples. HR-HPV: HR HPV genotypes. Non-HR-HPV: only non high risk HPV genotypes

Most patients who become HR-HPV-positive at follow-up reported a single HR positivity: 83.3% (5/6 patients) in case of negative result at baseline and 82.3% (14/17 patients) in the case of non-HR-HPV identification at baseline. A detailed description of the oral and anal HR-HPV patterns modifications from baseline to follow-up is reported in Fig. 3.

Fifty-nine subjects had a single type of HR-HPV found at baseline; at follow-up, 6 subjects (10.2%) became negative, 12 patients (20.3%) had non-HR-HPV and 41 (69.5%) had confirmed HR-HPV positivity. Twenty-four of the 41 patients had 1 HR-HPV strain at follow-up (16 subjects with a different genotype from baseline), and 17 (28.8%) subjects had an increased number of HR-HPV strains detected at follow-up. The percentage of MSM with confirmed HR-HPV detection at follow-up was 80% in the case of 2 HR found at baseline, 75% in the case of 3 HR-HPV found at baseline and 100% when 4 HR-HPV were found at baseline (only 3 patients were included in the subgroup with 4 HR genotypes); no patients with ≥3 types of HR-HPV at baseline had a negative result at follow-up (Table 2).

**Table 2:** Description of HR-HPV evolution from baseline to follow-up (24 months after baseline) according to the number of HR-HPV detected at baseline. The results obtained at follow-up are reported as absolute number and relative percentage within the specific subgroup

	Total number at baseline	Follow-up					
		Neg	non-HR	1 HR-HPV	2 HR-HPV	3 HR-HPV	≥4 HR-HPV
		n (%)	n (%)	n (%)	n (%)	n (%)	n (%)
1 HR-HPV at baseline	59	6 (10.2)	12 (20.3)	24 (40.7)	7 (11.9)	8 (13.5)	2 (3.4)
2 HR-HPV at baseline	30	2 (6.7)	4 (13.3)	9 (30)	4 (13.3)	6 (20)	5 (16.7)
3 HR-HPV at baseline	16	0	4 (25)	4 (25)	1 (6.3)	5 (31.2)	2 (12.5)
4 HR-HPV at baseline	3	0	0	0	2 (66.7)	1 (33.3)	0

HR: high risk HPV genotypes

non-HR: only not high risk HPV genotypes

HPV 16 was the most frequently detected genotype at both baseline and follow-up: no significant difference in the prevalence of the specific HR-HPV genotype at baseline and follow-up was found, and the percentages ranged from 3.03 to 16.97% at baseline and from 3.64 to 17.58% at follow-up. A concordant positivity of the same genotype at both time points was reported for HPV-16 (*p* < 0.001), HPV-18 (*p* < 0.001), HPV-31 (*p* = 0.000), HPV-35 (*p* = 0.05), HPV-45 (*p* = 0.000), HPV-51 (*p* = 0.000), HPV-58 (*p* = 0.004) and HPV-59 (*p* = 0.043).

### HPV infection by genotypes covered by the nonavalent vaccine

Overall, 7 of the 16 HPV infections detected in oral sites at baseline and follow-up were found to be caused by HR-HPV strains that were covered by the vaccine. Only 1 patient had HPV16 detected at both study times.

**Fig. 4:**
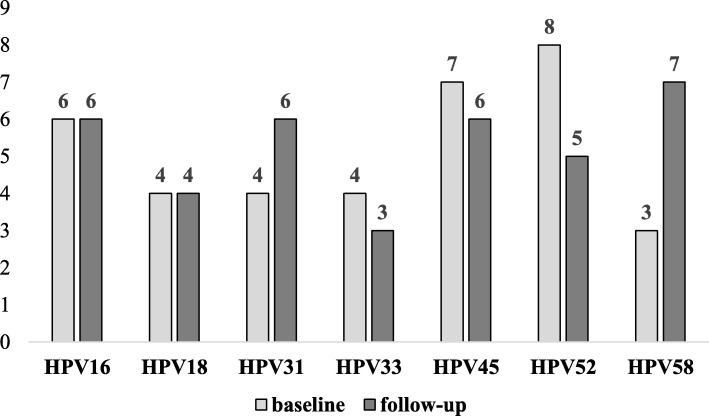
HR-HPV genotypes in the 25 patients who had only HR-HPV covered by the nonavalent vaccine both at baseline and at follow-up (24 months after baseline) in anal swabs. Data are expressed as absolute values. HR-HPV: high risk HPV genotypes. HPV: human papillomavirus

Nearly half of the patients with HR-HPV positivity at the anal site (54.6%, 59/108 patients at baseline and 50.5%, 52/103 patients at follow-up) had only HR-HPV genotypes covered by the vaccine, and 25 (15.2%) of the 165 subjects had this result at both study time points. The median age of the 25 patients was 44 years (range 39–55 years), and the nadir median value was 350 CD4+ cells/mm^3^ (range 26–535 CD4+ cells/mm^3^). Furthermore, single HR-HPV detection was the more frequent result (16 patients at baseline and 17 patients at follow-up). A detailed description of the HR-HPV genotypes identified at baseline and follow-up is reported in Fig. 4. Non-HR-HPV covered by the vaccine was not detected at the oral site while at anal site 5 patients were positive for HPV-6 (4 patients both at baseline and follow-up and one subject only at follow-up) and one patient was positive for HPV-11 at baseline and at follow-up.

## Discussion

This study focused on the characteristics of persistence and pattern evolution of HR-HPV infection in oral and anal areas over time in a cohort of HIV-positive MSM tested over a 24-month interval. We selected this study duration because the spontaneous clearance of nearly all HR-HPV strains occurs within this interval [16]; thus, we could identify the persistence of a specific HPV genotype and/or genotype patterns over time even if we cannot rule out possible reinfection.

In our opinion, HPV vaccination should be recommended for all HIV-positive MSM, regardless of age and of HPV detection at any site, even if published data are scarce, because of the high incidence of anal cancer in these subjects [17]. Subjects who remain HR-HPV-negative are the priority for vaccination, particularly if the negative result was confirmed over time and in more than one site, as in our small cohort of HIV-positive MSM (2.9% of all the patients included in this study). The European AIDS Clinical Society guidelines 2018 state that if HPV infection is established, the efficacy of the vaccine is questionable [10]; however, the role of a vaccine is to prevent infection, and HPV has the ability to be responsible for multiple infections. Therefore, the concept of established infection could potentially be applied to each HPV genotype. Furthermore, available HPV vaccines are safe and tolerable [18–20] and a persistent cross-reactive immune response against HR-HPV not covered by the vaccine was shown in vaccinated women [21]. However, vaccination has a clinically relevant role also in subjects with established HPV infection [13, 22]. We analysed the HR-HPV patterns in HIV-positive MSM retained in care in a tertiary level hospital of a high-income country that provides free care and therapy, which is the best clinical scenario. Based on our results, we believe that HPV vaccination should be prescribed in this clinical setting because all the tools that can reduce the risk of cancer should be used [23]. However, HPV vaccination prescription in different cohort of HIV+ MSM can be impeded by social and economical barriers [24].

The first result of our study was a significantly higher frequency of all HPV genotypes and of confirmed HR-HPV detection at the anal site at follow-up compared to the oral site (overall 81.8% versus 11.6 and 74.1% versus 40% for HR-HPV genotypes). Beachler et al. [25] reported the same difference in a cohort of 404 HIV subjects: (69 MSM): these data referred to any nononcogenic and oncogenic types; no specific analysis on HR was reported. Limited data concerning HR-HPV persistence in patients for whom more than a single site was tested have been reported. Parisi et al. [1] described a persistence rate of 71.4% in the anal site with no data regarding the oral site in 166 HIV+ MSM studied with a 6-month time interval between the 2 samplings. These authors demonstrated that any HR-HPV genotype detection in anal samples at either time point was positively related to a higher HIV plasma viral load and negatively related to ongoing ART. We investigated the role of plasma HIV viremia suppression on HR-HPV confirmed detection and found that the percentage of confirmed HR detection at both baseline and follow-up (2 years later) in this site was lower in patients receiving successful ART. The influence of ongoing plasma HIV viremia on HR-HPV detection over time has previously been studied with different approaches. Geskus et al. [26] described a lower HR-HPV clearance rate in MSM with higher plasma HIV RNA (relative to 1000 copies/ml) in a median time interval between measurements of 1.1 years, and Wiley et al. [27] demonstrated that the prevalence of HR-HPV was lower for every 10% increase in the number of study visits in which HIV RNA measured < 50 copies/mL during the study period. We could not evaluate the role of the HIV viral load in HR persistence at the oral site, possibly because of the low numerosity of the HR-HPV identified. The breakdown of the mucosal epithelial barrier by HIV may explain this association: Nazli et al. [28] demonstrated that exposure to HIV-1 led to an upregulation of inflammatory cytokine production and to a significant decrease in tight junction protein expression and increased permeability while Tugizov et al. [29] showed that tight junction disruption of the oral and anal epithelium due to the expression of the HIV tat and gp120 increased HPV pseudovirion paracellular penetration into the epithelium. However, we cannot exclude an independent role for immune-activation, as described for the interplay between HIV and CMV, EBV, and HHV-8 [30, 31].

In our study, the anal HR-HPV pattern was more complex than the oral pattern, both at baseline and at follow-up. The influence of the anatomic site of infection on the natural history of HPV is multifaceted and can include a faster clearance from oral epithelia and the continuous flow of saliva and associated immunoglobulins in the oral region [32, 33]. Papasavvas et al. [34] hypothesized a relationship between HR-HPV infection at the cervical site and increased immune activation in HIV infected women who were on successful ART, confirming the activating role of a viral co-infection previously reported for HCV in HIV patients with a virologic response [35]. This mechanism can contribute to the higher HR-HPV complexity (i.e., increased number of HR-HPV genotypes) at the anal site, which is rich in lymphoid tissue [36].

Almost all patients with HR-HPV positivity at both the anal and oral sites had different HR-HPV genotypes, as previously described [37, 38]. These results indicate the importance of testing a high-risk population in multiple sites: a single time point or single district data offered partial information both on HR-HPV detection (yes or no) and on possible multiple HPV detection in the specific patient. We found that only 2.9% of the subjects with oral and anal testing available were free from any HPV genotype and that 28.8% of the patients with one genotype of HR-HPV detected at baseline had an increased number of HR genotypes at follow-up. Based on this finding, we could speculate that almost all HIV+ MSM will become HPV positive during life and HPV detection could be missed or under evaluated with insufficient follow-up. Furthermore, two recent works [39, 40] described an association between multiple HPV infections and low-grade or high-grade squamous intraepithelial lesions at the anal site in HIV-positive men. Notably, the patients included in the work by Rovelli et al. [39] had no clinical symptoms. The presence of only HR-HPV genotypes covered by the nonavalent vaccine was demonstrated in nearly half of the patients at the oral and anal level. Therefore, an extended vaccination programme could reduce the increased complexity at the anal site and the risk of the first HR-HPV acquisition at the oral site [41].

During all DNA preparation, extraction and purification steps, precautions were taken to reduce the risk of false positive results or cross-contamination. However we cannot exclude a contamination. Besides this one, other limitations of this study must be recognized. First, we only sampled patients at 2 time points; thus, the evidence of persistent HR-HPV infection may instead be the clearance and subsequent transient exposure to the same genotype prior to the second sampling. Our HR-HPV detection was according to the type level; therefore, we could not exclude the possibility that someone could become infected with another variant of the same HR-HPV type; however, the young age of the subjects does not seem to indicate a modification in the exposure to risk. Second, only a small group of patients had all 4 samples available for testing, and positive HR detection was low at the oral site. These limitations restricted the power of the statistical analysis to detect correlations. Third, a more complete analysis on other sites of infection, such as the penis, and information about partners would make the analysis more informative. Fourth, we did not collect data on the patients’ sexual behaviour. Instead, our study focused on modifications of HR-HPV patterns after a 48-month study interval in adult patients rather than the reasons behind the modifications. Fifth, many oral samples were not evaluated because they were negative for beta globin, which indicates low cellularity; a different sampling method (i.e., toothbrush sampling) [42] could have helped increase the number of valid samples.

## Conclusions

In conclusion, HR-HPV detection is a clinical problem for almost all HIV positive MSM and requires careful surveillance. The anal HR-HPV pattern was more complex than the oral pattern at both baseline and follow-up. Effective ART seems to have a positive impact on HR-HPV confirmed detection at the anal level and should be associated with an extended vaccine campaign in light of ongoing new HIV diagnoses mostly in young patients [43, 44].

## Additional file

Additional file 1: Description of HR-HPV genotypes in oral and anal samples at baseline and at follow-up (24 months after baseline). Data are reported according to the number of HR-HPV genotypes detected and to the relative frequency of each specific combination within the group. (PDF 162 kb)

## Data Availability

The datasets generated and analysed for this study are not publicy available because they include clinical data.

## Abbreviations

ART: Antiretroviral therapy
HPV: Human papillomavirus
HR-HPV: High-risk HPV
MSM: Men who have sex with men
non-HR-HPV: Only not high risk HPV genotypess
PBS: Phosphate-buffered saline
vax-HPV: HR-HPV covered by the nonavalent HPV vaccine
