# Supplementary material for: Oral and anal high-risk human papilloma virus infection in HIV-positive men who have sex with men over a 24-month longitudinal study: complexity and vaccine implications
Source: BMC Public Health. 2019 May 28;19:645. doi: 10.1186/s12889-019-7004-x (PMC6537447; doi:10.1186/s12889-019-7004-x)
Supplement: Supplementary file 1 — Description of HR-HPV genotypes in oral and anal samples at baseline and at follow-up (24 months after baseline). Data are reported according to the number of HR-HPV genotypes detected and to the relative frequency of each specific combination within the group. (PDF 162 kb) [file 12889_2019_7004_MOESM1_ESM.pdf]

Additional file 1. Description of HR-HPV genotypes in oral and anal samples at baseline and at follow-up. Data are reported according to the number of HR-HPV genotypes detected and to the relative frequency of each specific combination within the group.

a) oral samples at baseline

HR-HPV genotype/s	Patients (n)	Percentage respect to the 106 patients tested	Relative frequency
1 HR-HPV detected (n=10)			
16	3	2.83	30
18	1	0.94	10
35	1	0.94	10
56	1	0.94	10
31	1	0.94	10
51	1	0.94	10
58	1	0.94	10
59	1	0.94	10

b) anal samples at baseline

HR-HPV genotype/s	Patients (n)	Percentage respect to the 165 patients tested	Relative frequency
1 HR-HPV detected (n=59)			
52	14	8.48	23.7
16	11	6.67	18.6
18	6	3.64	10.2
45	6	3.64	10.2
51	5	3.03	8.5
35	4	2.42	6.8
33	3	1.82	5.1
39	3	1.82	5.1
56	3	1.82	5.1
59	2	1.21	3.4
31	1	0.61	1.7
58	1	0.61	1.7
2 HR-HPV detected (n=30)			
16-45	3	1.82	10
16-18	2	1.21	6.7
16-51	2	1.21	6.7
18-31	2	1.21	6.7
18-51	2	1.21	6.7
33-58	2	1.21	6.7
39-51	2	1.21	6.7
51-52	2	1.21	6.7
16-31	1	0.61	3.3

18-52	1	0.61	3.3
18-56	1	0.61	3.3
31-45	1	0.61	3.3
31-51	1	0.61	3.3
31-52	1	0.61	3.3
31-58	1	0.61	3.3
33-39	1	0.61	3.3
39-45	1	0.61	3.3
45-51	1	0.61	3.3
45-56	1	0.61	3.3
51-59	1	0.61	3.3
52-56	1	0.61	3.3
3 HR-HPV detected (n=16)			
16-18-45	1	0.61	6.2
16-31-33	1	0.61	6.2
16-31-39	1	0.61	6.2
16-31-51	1	0.61	6.2
16-33-56	1	0.61	6.2
16-39-51	1	0.61	6.2
16-45-56	1	0.61	6.2
16-56-58	1	0.61	6.2
18-31-51	1	0.61	6.2
18-35-52	1	0.61	6.2
18-39-51	1	0.61	6.2
18-45-52	1	0.61	6.2
35-45-59	1	0.61	6.2
35-52-56	1	0.61	6.2
39-51-52	1	0.61	6.2
45-51-52	1	0.61	6.2
4 HR-HPV detected (n=3)			
16-31-35-59	1	0.61	33.3
18-31-33-56	1	0.61	33.3
18-51-52-56	1	0.61	33.3

c) oral samples at follow-up (24 months after baseline)

HR-HPV genotype	Patients (n)	Percentage respect to the 162 patients tested	Relative frequency
1 HR-HPV detected (n=11)			
16	3	1.8	27.3
56	2	1.2	18.2
59	2	1.2	18.2
33	1	0.6	9.1
35	1	0.6	9.1
45	1	0.6	9.1

58	1	0.6	9.1

d) anal samples at follow-up (24 months after baseline)

HR-HPV genotype/s	Patients (n)	Percentage respect to the 165 patients tested	Relative frequency
1 HR-HPV detected (n=56)			
45	10	6.06	17.8
52	8	4.85	14.3
16	7	4.24	12.5
59	6	3.64	10.7
33	5	3.03	8.9
51	5	3.03	8.9
58	5	3.03	8.9
31	3	1.81	5.3
18	2	1.21	3.6
35	2	1.21	3.6
39	2	1.21	3.6
56	1	0.61	1.8
2 HR-HPV detected (n=16)			
16-52	2	1.21	12.5
16-51	1	0.61	6.2
18-31	1	0.61	6.2
18-33	1	0.61	6.2
18-39	1	0.61	6.2
18-51	1	0.61	6.2
31-35	1	0.61	6.2
33-51	1	0.61	6.2
33-52	1	0.61	6.2
39-45	1	0.61	6.2
45-51	1	0.61	6.2
45-58	1	0.61	6.2
52-56	1	0.61	6.2
52-58	1	0.61	6.2
52-59	1	0.61	6.2
3 HR-HPV detected (n=21)			
16-31-39	2	1.21	9.5
16-51-52	2	1.21	9.5
18-51-52	2	1.21	9.5
16-18-31	1	0.61	4.8
16-18-58	1	0.61	4.8
16-31-33	1	0.61	4.8
16-31-52	1	0.61	4.8
16-31-59	1	0.61	4.8
16-39-51	1	0.61	4.8

16-51-58	1	0.61	4.8
16-52-59	1	0.61	4.8
18-33-35	1	0.61	4.8
18-52-59	1	0.61	4.8
31-33-51	1	0.61	4.8
31-33-56	1	0.61	4.8
31-51-56	1	0.61	4.8
31-52-58	1	0.61	4.8
35-51-52	1	0.61	4.8
4 HR-HPV detected (n=7)			
16-18-33-51	1	0.61	14.3
16-18-39-51	1	0.61	14.3
16-45-51-52	1	0.61	14.3
16-45-51-58	1	0.61	14.3
16-45-52-56	1	0.61	14.3
18-31-35-45	1	0.61	14.3
18-35-45-58	1	0.61	14.3
5 HR-HPV detected (n=3)			
16-31-35-52-59	1	0.61	33.3
16-33-52-56-59	1	0.61	33.3
18-31-51-52-58	1	0.61	33.3

HR-HPV: high risk HPV
HPV: Human papillomavirus
